# Effects of liraglutide on depressive behavior and cognitive function in the probe trial of Morris water maze test

**DOI:** 10.1192/j.eurpsy.2025.2083

**Published:** 2025-08-26

**Authors:** D.-I. Jon, J. G. Lee

**Affiliations:** 1 Hallym Univ. Sacred Heart Hospital, Anyang; 2 Haeundae Paik Hospital, Busan, Korea, Republic Of

## Abstract

**Introduction:**

Liraglutide, a glucagon-like peptide-1 (GLP-1) agonist, is an interesting candidate for improving metabolic syndrome and cognition in psychiatric disorders.

**Objectives:**

We investigated the effects of liraglutide on a depression-like phenotype in mice exposed to chronic unpredictable stress (CUS).

**Methods:**

Learning and memory were also assessed using the Morris water maze (MWM) test. Liraglutide (0.3 mg/kg/day for 21 days) was administered to mice with or without exposure to CUS. After 21 days of CUS, the forced swim test (FST) was performed to assess its antidepressant effect. To evaluate cognitive function, liraglutide was administered to mice under stress-free conditions for 21 days, and then the MWM test was performed on 6 consecutive days.

**Results:**

Chronic liraglutide treatment reduced FST immobility in mice with and without CUS. In the probe trial of the Morris water maze test, the search error rate was reduced and the time spent and path length in the target quadrant and the number of platform crossings were increased.

**Conclusions:**

Additional animal model experiments and molecular level studies are needed to support the results obtained in this study. Liraglutide appears to exert antidepressant effects and could improve cognitive function. Based on these results, GLP-1 agonists could have potential as novel antidepressants. It may also help with metabolic syndrome, cognitive dysfunction, and depressive symptoms.

**Disclosure of Interest:**

None Declared

